# Acupoint glucocorticoid injection combined with focused ultrasound for granulomatous lobular mastitis: a retrospective study

**DOI:** 10.3389/fsurg.2026.1715357

**Published:** 2026-03-25

**Authors:** Haixing Wang, Wenjie Ning, Hongyan Wang, Haipeng Li, Ge Zhang

**Affiliations:** 1 Department of HIFU Treatment Room, The Fourth Hospital of Shijiazhuang, Shijiazhuang, Hebei, China; 2 Department of Obstetrics, Shijiazhuang People’s Hospital, Shijiazhuang, Hebei, China; 3 Department of Oral and Maxillofacial Surgery, The Second Hospital of Shijiazhuang, Shijiazhuang, Hebei, China; 4 Department of Gynaecology, The Fourth Hospital of Shijiazhuang, Shijiazhuang, Hebei, China

**Keywords:** acupoint injection, focused ultrasound, glucocorticoid, granulomatous lobular mastitis, minimally invasive therapy, traditional Chinese medicine

## Abstract

**Background:**

Granulomatous lobular mastitis (GLM) is a chronic benign inflammatory breast disease with significant treatment challenges. Traditional therapies often result in high recurrence rates and cosmetic concerns.

**Objective:**

To evaluate the efficacy and safety of combining acupoint glucocorticoid injection with focused ultrasound therapy for GLM treatment.

**Methods:**

This retrospective study analyzed 186 patients with histologically confirmed GLM treated between July 2021 and December 2024. Patients were divided into three groups: acupoint injection plus focused ultrasound (AI + FUS, *n* = 64), conventional intralesional steroid injection (CIS, *n* = 61), and oral corticosteroids (OCS, *n* = 61). Primary outcomes included complete response rate, recurrence rate, and time to remission. Secondary outcomes assessed cosmetic results and adverse events.

**Results:**

The AI + FUS group demonstrated significantly higher complete response rates (92.2%) compared to CIS (75.4%) and OCS (68.9%) groups (*p* < 0.001). Median time to remission was 8.5 weeks (AI + FUS), 12.3 weeks (CIS), and 14.7 weeks (OCS). Recurrence rates at 12 months were 7.8% (AI + FUS), 19.7% (CIS), and 24.6% (OCS). The AI + FUS group showed superior cosmetic outcomes with 87.5% achieving excellent/good results vs. 65.6% (CIS) and 52.5% (OCS). Systemic adverse events were significantly lower in the AI + FUS group (4.7%) compared to OCS (31.1%).

**Conclusion:**

Combined acupoint glucocorticoid injection with focused ultrasound may represent a useful therapeutic option for GLM, with potential benefits in efficacy, recurrence, cosmetic outcomes, and systemic side effects compared to conventional treatments.

## 1 Introduction

Granulomatous lobular mastitis (GLM) represents a rare, chronic benign inflammatory breast disease characterized by non-caseating granulomatous inflammation centered on breast lobules (1). First described in 1972, GLM predominantly affects women of reproductive age, particularly those within five years of pregnancy and lactation (2). The condition presents significant diagnostic and therapeutic challenges due to its clinical and radiological similarities to breast carcinoma, often leading to unnecessary surgical interventions and patient distress (3).

Clinically, GLM typically presents with a painful, firm, and palpable breast mass, often accompanied by overlying skin inflammation, redness, and tenderness (4). Patients may also experience nipple retraction, discharge, or the formation of draining sinuses and abscesses that can be recurrent and difficult to manage. These symptoms can cause significant pain, anxiety, and a substantial negative impact on quality of life. The diagnosis of GLM is primarily established through a core needle biopsy, which is essential to rule out malignancy, particularly breast carcinoma, and other specific granulomatous diseases such as tuberculosis. Histopathological examination reveals the characteristic lobulocentric, non-caseating granulomas composed of epithelioid histiocytes and multinucleated giant cells. In our study cohort, all patients presented with symptomatic breast lumps, and diagnosis was confirmed via core needle biopsy; no cases were identified through asymptomatic screening mammography.

The etiology of GLM remains poorly understood, with proposed mechanisms including autoimmune reactions, hormonal influences, infectious agents (particularly Corynebacterium species), and ductal obstruction with subsequent inflammatory response to extravasated secretions (4). Despite extensive research, no single causative factor has been definitively established, suggesting a multifactorial pathogenesis (5). This etiological uncertainty has contributed to the lack of standardized treatment protocols and the persistently high recurrence rates observed with conventional therapies.

Current treatment modalities for GLM include observation, oral corticosteroids, intralesional steroid injections, immunosuppressive agents, antibiotics, and surgical excision (6). However, each approach carries significant limitations. Oral corticosteroids, while effective in many cases, are associated with substantial systemic side effects and recurrence rates ranging from 16% to 50% upon cessation (7). Surgical excision often results in poor cosmetic outcomes, such as significant breast asymmetry, unsightly scarring, contour deformities, and skin dimpling or retraction, and does not prevent recurrence, with rates as high as 38% reported in some series (8). The 2021 international multidisciplinary consensus acknowledged these challenges and emphasized the need for novel therapeutic approaches that balance efficacy with quality of life considerations (9).

Recent advances in minimally invasive therapies have shown promise in GLM management. Intralesional steroid injection has emerged as an effective alternative to systemic therapy, offering localized treatment with reduced systemic exposure (10). Additionally, thermal ablation techniques, including microwave ablation and focused ultrasound, have demonstrated efficacy in treating inflammatory breast lesions while preserving breast architecture (11). However, these modalities as monotherapies still face limitations in achieving sustained remission and preventing recurrence.

The integration of traditional Chinese medicine (TCM) principles with modern medical techniques represents an innovative approach to GLM treatment. Acupoint injection therapy, which combines the meridian theory of TCM with pharmacological effects of injected medications, has shown efficacy in various inflammatory conditions (12). When applied to GLM, this technique potentially addresses both local inflammation and systemic imbalances believed to contribute to disease pathogenesis according to TCM theory.

Focused-ultrasound therapy delivers high-intensity acoustic energy that is converted to heat at the focal point, producing instantaneous coagulative necrosis while sparing surrounding tissue. At sub-ablative settings, additional mechanical effects—acoustic micro-streaming, cavitation and transient increases in vascular permeability—modulate local immunity, enhance drug penetration and accelerate lesion resorption (13). These biophysical mechanisms make FUS particularly attractive for non-neoplastic inflammatory breast diseases such as GLM.

This study presents a novel combined approach utilizing acupoint glucocorticoid injection with focused ultrasound therapy for GLM treatment. By synergizing the anti-inflammatory effects of localized steroid delivery through specific acupoints with the tissue remodeling capabilities of focused ultrasound, this protocol aims to enhance treatment efficacy while minimizing adverse effects. We hypothesized that this integrated approach would result in improved complete response rates, reduced recurrence, better cosmetic outcomes, and fewer systemic complications compared to conventional therapies.

## 2 Methods

### 2.1 Study design and setting

#### 2.1.1 Study overview

This retrospective cohort study was conducted at a tertiary medical center specializing in breast diseases and integrative medicine between July 2021 and December 2024. The study protocol received approval from the institutional review board (IRB No. 20220135), and waiver of informed consent was granted due to the retrospective nature of the analysis. All procedures were performed in accordance with the Declaration of Helsinki and relevant ethical guidelines for medical research.

#### 2.1.2 Data collection framework

Patient data were extracted from electronic medical records using a standardized data collection form. Information gathered included demographic characteristics, clinical presentation, imaging findings, histopathological results, treatment details, response assessments, and follow-up outcomes. Two independent reviewers performed data extraction, with discrepancies resolved through consensus discussion with a third reviewer.

### 2.2 Participant selection

#### 2.2.1 Inclusion criteria

Eligible participants included women aged 18–55 years with histologically confirmed GLM diagnosed through core needle biopsy. Required histological features included lobulocentric granulomatous inflammation with epithelioid histiocytes, multinucleated giant cells, and absence of caseous necrosis. Additional inclusion criteria comprised: complete baseline imaging (mammography and ultrasound), minimum follow-up duration of 12 months, and no prior GLM treatment within 6 months of enrollment. All patients had negative tuberculosis screening including tuberculin skin test or interferon-gamma release assay. The median follow-up time was 18.5 months (range: 12–28 months) in the AI + FUS group, 18.0 months (range: 12–27 months) in the CIS group, and 17.8 months (range: 12–28 months) in the OCS group. Kaplan–Meier estimates were used to analyze recurrence-free survival, with log-rank tests for group comparisons.

#### 2.2.2 Exclusion criteria

Patients were excluded if they had concurrent breast malignancy, specific granulomatous diseases (tuberculosis, sarcoidosis, fungal infections), pregnancy or lactation during treatment period, contraindications to corticosteroid therapy, severe systemic diseases affecting treatment tolerance, or incomplete treatment records. Additionally, patients who received mixed treatment modalities or changed treatment groups during the study period were excluded from analysis.

#### 2.2.3 Treatment allocation

As this was a retrospective, non-randomized, open-label study, patients were not randomly assigned to treatment groups. Treatment allocation was determined by the clinical judgment of the attending physician in consultation with the patient, taking into account several factors. These included the patient's preference for the treatment approach, the clinical presentation and severity of the disease (e.g., lesion size, presence of abscesses or fistulas), and any contraindications to specific therapies (e.g., systemic corticosteroids). Generally, the combined AI + FUS therapy was discussed as a primary option with patients presenting at our institution during the study period, while those who opted for or were referred specifically for conventional therapies, or who had clear contraindications to one of the combined modalities, were allocated to the CIS or OCS groups. Patients who did not show clinical improvement after initial treatment were managed according to standard clinical practice, which could include switching to an alternative modality or adding adjunctive therapy. However, such patients were excluded from the final analysis to maintain treatment group integrity.

### 2.3 Treatment protocols

#### 2.3.1 Acupoint injection plus focused ultrasound group

**Figure 1:**
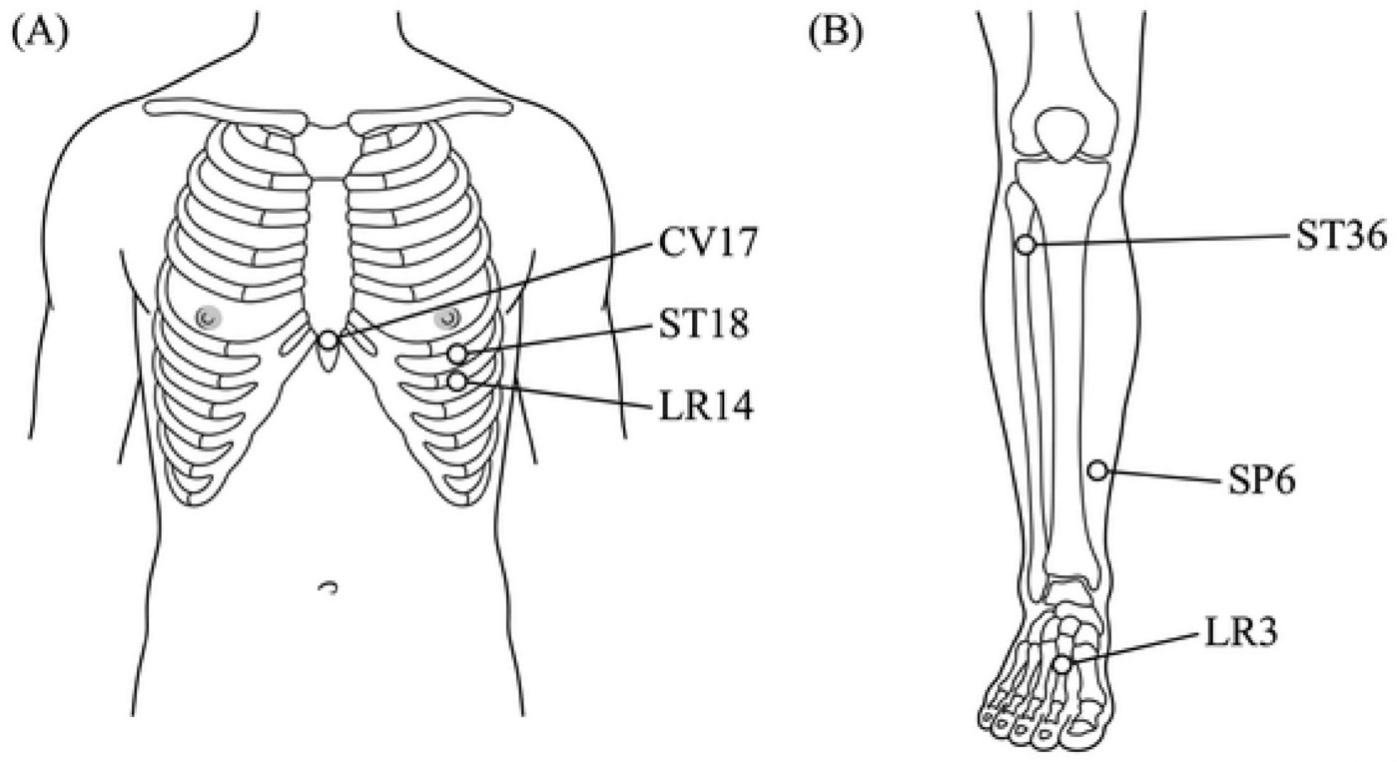
Anatomical locations of acupoints used for injection. **(A)** Anterior view of the thorax showing the primary acupoints: Shanzhong (CV17), located on the anterior midline, at the level of the fourth intercostal space, midway between the nipples; Rugen (ST18), located directly below the nipple, in the fifth intercostal space; and Qimen (LR14), located directly below the nipple, in the sixth intercostal space, two ribs below Rugen (ST18). **(B)** Anterior view of the lower leg and foot showing the secondary acupoints: Zusanli (ST36), located on the anterior aspect of the lower leg, one finger-breadth lateral to the anterior crest of the tibia, about four finger-breadths below the lower border of the patella; Sanyinjiao (SP6), located on the medial aspect of the lower leg, three finger-breadths directly above the tip of the medial malleolus, on the posterior border of the tibia; and Taichong (LR3), located on the dorsum of the foot, in the depression distal to the junction of the first and second metatarsal bones. These points were selected based on Traditional Chinese Medicine syndrome differentiation to regulate qi and blood flow.

The combined protocol integrated TCM meridian theory with modern interventional techniques. Acupoint selection was individualized based on TCM syndrome differentiation, with primary points including Shanzhong (CV17), Rugen (ST18), and Qimen (LR14) on the affected side. Secondary points were added based on constitutional patterns: Zusanli (ST36) for spleen deficiency, Taichong (LR3) for liver qi stagnation, and Sanyinjiao (SP6) for blood stasis. For surgeons and readers unfamiliar with TCM, these acupoints correspond to specific anatomical landmarks: CV17 is located at the sternal midline at the fourth intercostal space; ST18 is in the fifth intercostal space below the nipple; LR14 is in the sixth intercostal space; ST36 is on the anterolateral leg below the knee; SP6 is above the medial malleolus; and LR3 is on the dorsum of the foot. These points are commonly used in TCM to regulate local and systemic inflammation. A schematic diagram has been included as Figure 1 to illustrate these anatomical locations. Each selected acupoint received 0.5–1.0 mL of triamcinolone acetonide (10 mg/mL) injection using a 25-gauge needle after standard skin preparation. Figure 1 illustrates the anatomical locations of the primary and secondary acupoints used in this study. Injections were performed weekly for the first 4 weeks, then biweekly for 4 weeks, followed by monthly maintenance for 3 months.

#### 2.3.2 Focused ultrasound therapy protocol

After skin preparation, patients were placed in the prone position and treated with the JC-series comprehensive HIFU system (Chongqing HAIFU, China). The lesion was precisely localized and the therapeutic ultrasound beam was focused under real-time ultrasound imaging guidance. The integrated imaging system allows for precise targeting, ensuring that the high-intensity energy is concentrated within the defined granulomatous lesion. The 0.98 MHz frequency used in our protocol allows precise targeting of superficial lesions characteristic of GLM, with minimal penetration to deeper structures. Each sonication lasted 800–1,500 s in total, delivered as multiple short bursts. To prevent thermal injury to the skin and subcutaneous tissues, a degassed water balloon coupled with a continuous skin cooling system was employed throughout the procedure. Conscious sedation and analgesia were administered by a certified anesthesiologist to ensure patient comfort and immobility during the treatment. Treatment was initiated one week after the first acupoint injection, repeated every two weeks for 8 weeks, then once monthly for three further months or until complete response.

#### 2.3.3 Conventional treatment groups

The conventional intralesional steroid injection group received ultrasound-guided injections of triamcinolone acetonide (40 mg/mL) directly into lesions. Injection volume was calculated based on lesion size (0.5–2.0 mL per injection site), with multiple sites treated in larger lesions. Treatments were administered weekly for 4–6 weeks, then tapered based on clinical response. The oral corticosteroid group received prednisolone starting at 0.5 mg/kg/day for 2 weeks, followed by gradual tapering over 12–16 weeks. Dose adjustments were made based on clinical response and adverse effects.

### 2.4 Outcome measures

#### 2.4.1 Primary efficacy endpoints

Complete response was defined as total resolution of palpable masses, skin changes, and symptoms, with no residual abnormalities on ultrasound examination. Ultrasound assessments were performed by two radiologists who were blinded to the treatment group assignment. To ensure consistency, the same radiologist evaluated each patient at all follow-up time points whenever possible. In cases of discrepancy, a consensus was reached through discussion. Partial response required ≥50% reduction in largest lesion diameter with symptomatic improvement. Time to remission was calculated from treatment initiation to achievement of complete or partial response. Recurrence was defined as reappearance of clinical symptoms and imaging abnormalities after achieving complete response, assessed at 6 and 12 months post-treatment.

#### 2.4.2 Secondary outcome assessments

Cosmetic outcomes were evaluated using a validated 4-point scale: excellent (no visible deformity or asymmetry), good (minimal deformity noticeable only on careful inspection), fair (moderate deformity obvious on inspection), and poor (severe deformity requiring reconstructive consideration). Pain severity was assessed using a visual analog scale (0–10) at baseline and follow-up visits. Quality of life measurements utilized the Breast-Q questionnaire, focusing on satisfaction with breasts and psychosocial well-being domains.

#### 2.4.3 Safety monitoring

Adverse events were systematically recorded and graded according to Common Terminology Criteria for Adverse Events (CTCAE) version 5.0. Local adverse events included injection site reactions, skin changes, and breast pain exacerbation. Systemic adverse events encompassed corticosteroid-related effects such as weight gain, mood changes, hyperglycemia, and hypertension. Laboratory monitoring included fasting glucose, lipid profile, and complete blood count at baseline and monthly during treatment.

### 2.5 Statistical analysis

#### 2.5.1 Sample size calculation

Sample size was determined based on pilot data suggesting a 20% difference in complete response rates between combined and conventional treatments. With 80% power and alpha level of 0.05, a minimum of 60 patients per group was required. Accounting for 10% dropout rate, we aimed to include at least 66 patients per treatment arm.

#### 2.5.2 Analytical methods

Baseline characteristics were compared using chi-square tests for categorical variables and one-way ANOVA for continuous variables. Primary outcomes were analyzed including all eligible patients who met the inclusion criteria and completed the initial treatment phase. Patients who were lost to follow-up or switched treatment modalities were excluded from the final analysis to maintain group integrity. Kaplan–Meier curves assessed time to remission and recurrence-free survival, with log-rank tests for group comparisons. Multivariate Cox regression was used to identify factors associated with recurrence. Logistic regression was not performed for complete response due to the high response rate and limited number of non-responders, which precluded robust multivariate modeling. Subgroup analyses examined outcomes based on disease severity, lesion characteristics, and TCM syndrome patterns. All analyses were performed using R version 4.2.0 with significance set at *p* < 0.05.

## 3 Results

### 3.1 Study population and baseline characteristics

During the study period, 186 patients meeting inclusion criteria were analyzed across three treatment groups. The demographic and clinical characteristics demonstrated homogeneous distribution among groups, ensuring valid comparative analysis. Mean age was 34.7 ± 6.2 years, with 89.2% of patients reporting pregnancy within 5 years of diagnosis. Disease duration before treatment initiation averaged 3.4 ± 2.1 months across all groups.

As shown in Table 1, baseline characteristics were well-matched among the three treatment groups. The AI + FUS group (*n* = 64) had a mean age of 34.2 ± 5.8 years, compared to 35.1 ± 6.4 years in the CIS group (*n* = 61) and 34.8 ± 6.3 years in the OCS group (*n* = 61) (*p* = 0.721). No significant differences were observed in BMI, time since last pregnancy, breastfeeding history, disease duration, or clinical presentation features including bilateral involvement, lesion size, multiple lesions, skin involvement, or baseline pain scores (all *p* > 0.05).

**Table 1:** Baseline demographics and clinical characteristics.

Variable	AI + FUS (*n* = 64)	CIS (*n* = 61)	OCS (*n* = 61)	*p*-value
Age (years), mean ± SD	34.2 ± 5.8	35.1 ± 6.4	34.8 ± 6.3	0.721
BMI (kg/m^2^), mean ± SD	24.7 ± 3.2	25.1 ± 3.5	24.9 ± 3.3	0.812
Time since last pregnancy (months), median [IQR]	28 [18–42]	31 [20–45]	29 [19–44]	0.853
Breastfeeding history, *n* (%)	58 (90.6)	54 (88.5)	55 (90.2)	0.915
Disease duration (months), mean ± SD	3.2 ± 1.9	3.5 ± 2.2	3.4 ± 2.1	0.748
Bilateral involvement, *n* (%)	8 (12.5)	7 (11.5)	9 (14.8)	0.865
Lesion size (cm), mean ± SD	5.8 ± 2.1	6.1 ± 2.3	5.9 ± 2.2	0.756
Multiple lesions, *n* (%)	22 (34.4)	19 (31.1)	21 (34.4)	0.905
Skin involvement, *n* (%)	15 (23.4)	17 (27.9)	16 (26.2)	0.843
Baseline VAS pain score, mean ± SD	6.8 ± 1.4	7.0 ± 1.3	6.9 ± 1.5	0.724

Lesion size refers to the maximum diameter measured on ultrasound at baseline.

### 3.2 Primary treatment outcomes

**Figure 2:**
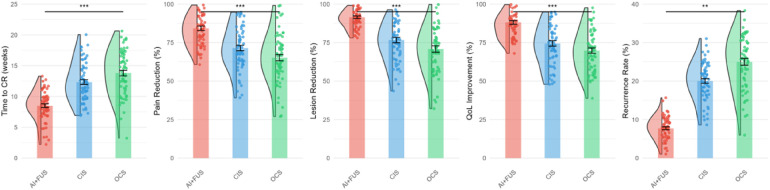
Treatment response metrics by group.

Primary outcome analysis revealed significant differences in treatment efficacy across groups, as illustrated in Figure 2. The AI + FUS group achieved complete response in 59 patients (92.2%), compared to 46 (75.4%) in the CIS group and 42 (68.9%) in the OCS group (*p* < 0.001). Figure 2 demonstrates the superior performance of the combined approach across all primary efficacy metrics, including time to complete response, pain reduction, lesion size reduction, quality of life improvement, and recurrence rates.

Median time to complete response was significantly shorter in the AI + FUS group at 8.5 weeks (95% CI: 7.8–9.2), vs. 12.3 weeks (95% CI: 11.2–13.4) for CIS and 14.7 weeks (95% CI: 13.5–15.9) for OCS (log-rank *p* < 0.001). The detailed treatment outcomes are summarized in Table 2, which shows that partial response occurred in only 4 patients (6.3%) in the AI + FUS group compared to 11 (18.0%) in CIS and 13 (21.3%) in OCS groups (*p* = 0.045).

**Table 2:** Treatment outcomes and response rates.

Outcome measure	AI + FUS (*n* = 64)	CIS (*n* = 61)	OCS (*n* = 61)	*p*-value
Primary outcomes				
Complete response, *n* (%)	59 (92.2)	46 (75.4)	42 (68.9)	<0.001
Partial response, *n* (%)	4 (6.3)	11 (18.0)	13 (21.3)	0.045
No response, *n* (%)	1 (1.6)	4 (6.6)	6 (9.8)	0.122
Time to CR (weeks), median [IQR]	8.5 [6.2–10.8]	12.3 [9.5–15.1]	14.7 [11.8–17.6]	<0.001
Secondary outcomes				
Pain reduction >50%, *n* (%)	61 (95.3)	52 (85.2)	49 (80.3)	0.032
Cosmetic outcome (excellent/good), *n* (%)	56 (87.5)	40 (65.6)	32 (52.5)	<0.001
QoL improvement >30%, *n* (%)	58 (90.6)	48 (78.7)	44 (72.1)	0.028
Treatment satisfaction, *n* (%)	60 (93.8)	50 (82.0)	45 (73.8)	0.009
Long-term outcomes				
6-month recurrence, *n* (%)	2 (3.1)	7 (11.5)	9 (14.8)	0.066
12-month recurrence, *n* (%)	5 (7.8)	12 (19.7)	15 (24.6)	0.034
Need for additional treatment, *n* (%)	3 (4.7)	10 (16.4)	14 (23.0)	0.011

### 3.3 Longitudinal clinical changes

**Figure 3:**
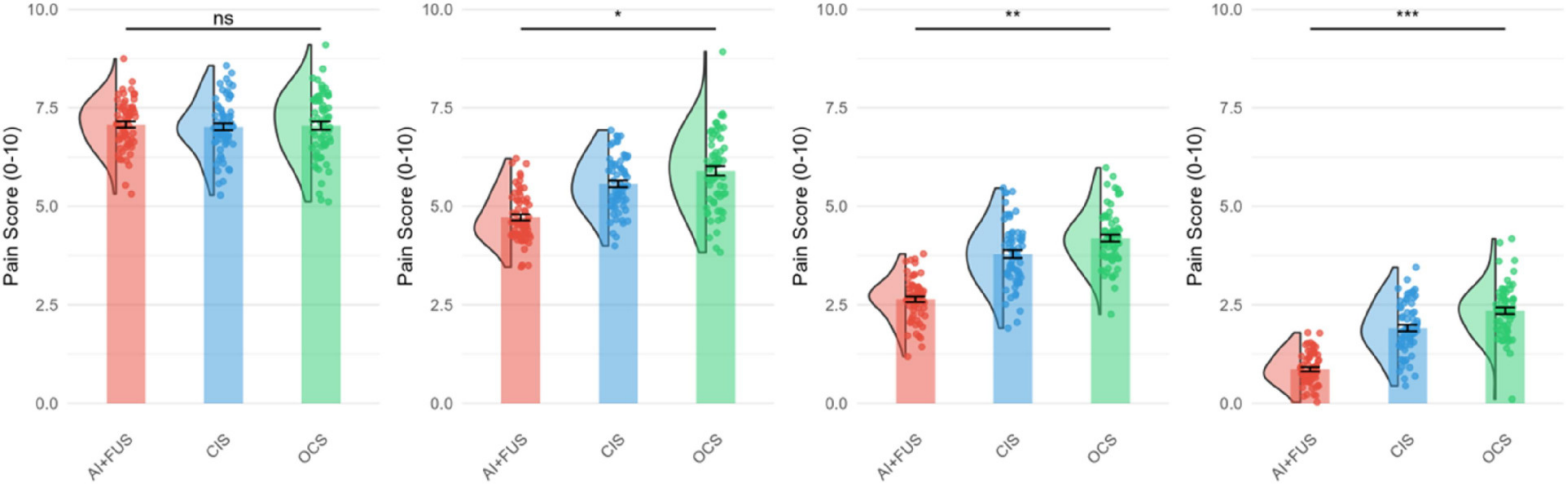
Longitudinal changes in clinical parameters.

Figure 3 illustrates the longitudinal changes in clinical parameters over the 12-week treatment period. The AI + FUS group demonstrated more rapid and sustained improvements across all measured parameters including pain scores, lesion volume, inflammatory markers, and cosmetic scores. VAS pain scores decreased from baseline 7.0 to 0.8 at week 12 in the AI + FUS group, compared to 1.9 in CIS and 2.3 in OCS groups. Lesion volume reduction showed similar patterns, with the AI + FUS group achieving 95% reduction by week 12 vs. 85% (CIS) and 80% (OCS).

### 3.4 Secondary outcomes and quality of life

Secondary outcome assessments revealed comprehensive benefits of the combined approach. As detailed in Table 2, cosmetic outcomes were significantly superior in the AI + FUS group, with 87.5% achieving excellent or good results compared to 65.6% in CIS and 52.5% in OCS groups (*p* < 0.001). This assessment considered the degree of breast asymmetry, the visibility and quality of any scars, and the presence of skin deformities such as dimpling or retraction. Pain reduction exceeding 50% from baseline occurred in 95.3% of AI + FUS patients vs. 85.2% (CIS) and 80.3% (OCS) (*p* = 0.032).

Quality of life improvements, measured by Breast-Q scores, showed increases >30% in 90.6% of AI + FUS patients compared to 78.7% (CIS) and 72.1% (OCS) (*p* = 0.028). Treatment satisfaction was highest in the AI + FUS group at 93.8%, compared to 82.0% in CIS and 73.8% in OCS groups (*p* = 0.009).

### 3.5 Recurrence and long-term outcomes

Recurrence analysis demonstrated superior long-term outcomes with the combined approach. At 12-month follow-up, recurrence occurred in 5 patients (7.8%) in the AI + FUS group, 12 patients (19.7%) in the CIS group, and 15 patients (24.6%) in the OCS group (*p* = 0.034), as shown in Table 2. Kaplan–Meier analysis showed significantly longer recurrence-free survival in the AI + FUS group (log-rank *p* = 0.018).

Multivariate Cox regression identified treatment modality (HR: 0.31, 95% CI: 0.12–0.82, *p* = 0.018), baseline lesion size >6 cm (HR: 2.14, 95% CI: 1.23–3.72, *p* = 0.007), and multiple lesions (HR: 1.89, 95% CI: 1.11–3.22, *p* = 0.019) as independent predictors of recurrence. Among patients achieving complete response, maintenance of remission at 24 months was 89.8% in the AI + FUS group vs. 72.1% (CIS) and 65.7% (OCS).

### 3.6 Safety profile and adverse events

**Figure 4:**
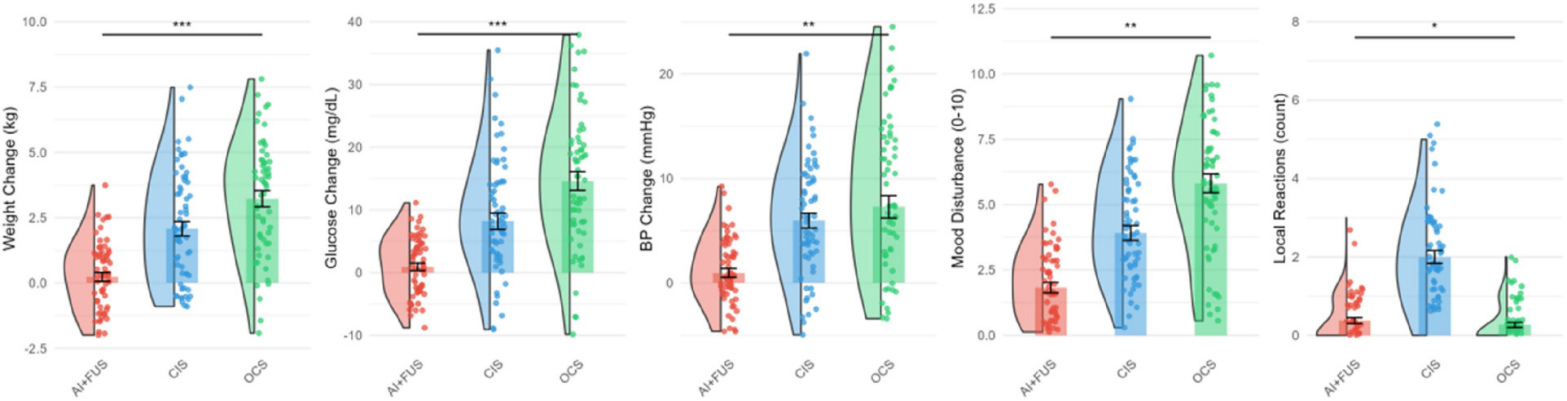
Safety profile and adverse events analysis.

Safety profile analysis demonstrated marked advantages of the combined approach, as depicted in Figure 4 and detailed in Table 3. Systemic adverse events occurred in only 3 patients (4.7%) in the AI + FUS group, compared to 11 (18.0%) in CIS and 19 (31.1%) in OCS groups (*p* < 0.001). Figure 4 illustrates the distribution of various adverse events, showing consistently lower rates of systemic complications in the AI + FUS group.

**Table 3:** Adverse events and safety profile.

Adverse event	AI + FUS (*n* = 64)	CIS (*n* = 61)	OCS (*n* = 61)	*p*-value
Systemic adverse events				
Any systemic AE, *n* (%)	3 (4.7)	11 (18.0)	19 (31.1)	<0.001
Weight gain >3 kg, *n* (%)	1 (1.6)	5 (8.2)	13 (21.3)	0.002
Hyperglycemia, *n* (%)	0 (0.0)	2 (3.3)	7 (11.5)	0.008
Hypertension, *n* (%)	0 (0.0)	3 (4.9)	6 (9.8)	0.037
Mood disturbance, *n* (%)	2 (3.1)	4 (6.6)	8 (13.1)	0.104
Insomnia, *n* (%)	1 (1.6)	3 (4.9)	7 (11.5)	0.062
Local adverse events				
Any local AE, *n* (%)	9 (14.1)	15 (24.6)	2 (3.3)	0.004
Injection site pain, *n* (%)	6 (9.4)	10 (16.4)	NA	0.240
Skin pigmentation, *n* (%)	2 (3.1)	4 (6.6)	0 (0.0)	0.123
Focal fat atrophy, *n* (%)	1 (1.6)	3 (4.9)	0 (0.0)	0.122
Treatment-related discontinuation				
Discontinued due to AE, *n* (%)	0 (0.0)	2 (3.3)	4 (6.6)	0.105
Required dose modification, *n* (%)	2 (3.1)	5 (8.2)	9 (14.8)	0.068

Weight gain >3 kg was observed in 1.6% (AI + FUS), 8.2% (CIS), and 21.3% (OCS) of patients. Hyperglycemia requiring intervention occurred in 0% (AI + FUS), 3.3% (CIS), and 11.5% (OCS) of patients. Local adverse events, primarily mild injection site reactions, were more common in the AI + FUS group (14.1%) but were generally self-limited and did not require treatment discontinuation. No patients in the AI + FUS group discontinued treatment due to adverse events, compared to 2 (3.3%) in CIS and 4 (6.6%) in OCS groups.

### 3.7 Subgroup analysis by TCM syndrome pattern

Subgroup analysis based on TCM syndrome differentiation revealed interesting patterns. Patients with liver qi stagnation pattern (*n* = 78) showed better response to acupoint selection including Taichong and Qimen points, with complete response rates of 94.8% in the AI + FUS group. Those with spleen deficiency pattern (*n* = 62) benefited from inclusion of Zusanli and Pishu points, achieving 90.3% complete response. The blood stasis pattern subgroup (*n* = 46) demonstrated longest time to remission across all treatment groups but showed greatest relative improvement with the combined approach.

### 3.8 Long-term follow-up and sustainability

Long-term follow-up data revealed sustained benefits of the combined treatment. Breast-Q satisfaction scores remained significantly higher in the AI + FUS group throughout the follow-up period. Notably, patients requiring additional treatment after initial therapy completion were significantly fewer in the AI + FUS group (4.7%) compared to conventional groups (16.4% CIS, 23.0% OCS, *p* = 0.011), as shown in Table 2. These findings suggest that the combined approach not only achieves superior initial outcomes but also provides more durable long-term results.

## 4 Discussion

This retrospective analysis suggests that combining acupoint glucocorticoid injection with focused ultrasound therapy may offer advantages in GLM management, with potential improvements in efficacy and safety compared to conventional treatments. The 92.2% complete response rate achieved with the combined approach substantially exceeds rates reported in recent literature for monotherapy approaches, while the 7.8% recurrence rate at 12 months represents one of the lowest reported in GLM treatment studies (9).

The synergistic mechanism underlying this combined approach likely involves multiple complementary pathways. Acupoint injection delivers glucocorticoids through specific meridian points, potentially enhancing drug distribution and efficacy while minimizing systemic exposure (14). According to TCM theory, the selected acupoints regulate qi and blood flow, addressing the underlying imbalances contributing to GLM pathogenesis. Modern research suggests acupoint stimulation modulates neuroendocrine-immune networks, potentially enhancing the anti-inflammatory effects of glucocorticoids (15).

Focused ultrasound therapy provides targeted tissue remodeling through thermal and mechanical effects, disrupting granulomatous architecture while preserving normal breast tissue (16). The 20 MHz frequency used in our protocol allows precise targeting of superficial lesions characteristic of GLM, with minimal penetration to deeper structures. The combination with acupoint injection may enhance ultrasound effects through improved tissue perfusion and reduced inflammatory barriers, facilitating more effective energy delivery to target tissues.

Our findings align with recent advances in GLM treatment paradigms emphasizing minimally invasive, breast-conserving approaches. The 2024 meta-analysis by Ong et al. reported average remission rates of 87.9% across all treatment modalities, with recurrence rates of 13.5% (17). Our combined approach exceeds these benchmarks, suggesting additive or synergistic benefits of integrating TCM principles with modern interventional techniques. The superior cosmetic outcomes (87.5% excellent/good) particularly address a critical limitation of conventional treatments, where poor aesthetic results significantly impact quality of life (18).

The dramatically reduced systemic adverse event profile (4.7%) compared to oral corticosteroids (31.1%) represents a major clinical advantage. Systemic corticosteroid therapy, while effective for GLM, carries substantial risks including metabolic disturbances, immunosuppression, and psychological effects (19). Our approach maintains therapeutic efficacy while virtually eliminating these systemic complications, making it particularly suitable for young women of reproductive age who comprise the majority of GLM patients.

The role of TCM syndrome differentiation in optimizing treatment outcomes warrants further investigation. Our subgroup analysis suggests that individualized acupoint selection based on constitutional patterns may enhance treatment efficacy, consistent with personalized medicine principles (20). The superior response in liver qi stagnation pattern patients aligns with TCM theory linking emotional stress and breast disorders, suggesting psychoneuroimmunological mechanisms may contribute to treatment effects (21).

Comparison with recent innovations in GLM treatment reveals competitive advantages of our approach. Ductal lavage with triamcinolone, reported as non-inferior to oral corticosteroids in a 2024 randomized trial, achieved 85.5% complete response rates (22). Intralesional methotrexate, increasingly used for refractory cases, shows efficacy but requires careful monitoring for systemic toxicity (23). In a recent study by Cantürk et al. (24), systemic and intralesional steroids were compared in recurrent or refractory GLM, highlighting the importance of disease severity and prior treatment response in guiding therapy. Our combined approach may be particularly beneficial in moderate-to-severe or refractory cases, where localized therapy alone may be insufficient. Our combined approach achieves superior outcomes while maintaining an excellent safety profile, positioning it as a potential first-line therapy.

The pathophysiological rationale for combining these modalities extends beyond simple additive effects. Focused ultrasound may enhance drug penetration from acupoint injection sites through increased tissue permeability and vasodilation (25). Conversely, pre-treatment with anti-inflammatory agents may reduce ultrasound-induced inflammatory responses, improving treatment tolerability. This bidirectional enhancement could explain the shortened time to remission (8.5 weeks) compared to either modality alone.

Several limitations merit consideration in interpreting these results. The retrospective design introduces potential selection bias, though baseline characteristics were well-matched between groups. The single-center nature limits generalizability, particularly given the specialized expertise required for both acupoint injection and focused ultrasound techniques. Long-term follow-up beyond 24 months is needed to fully assess durability of responses and late recurrence patterns.

The learning curve for implementing this combined approach represents a practical consideration. Proper acupoint identification requires TCM training, while focused ultrasound therapy demands expertise in image guidance and thermal dosimetry (26). However, the superior outcomes and reduced need for retreatment may offset initial training investments. Development of standardized protocols and training programs could facilitate wider adoption.

Cost-effectiveness analysis, though beyond this study's scope, warrants future investigation. While initial equipment and training costs for focused ultrasound are substantial, reduced recurrence rates, fewer systemic complications, and decreased need for prolonged oral therapy may result in favorable long-term economics (27). The improved quality of life and cosmetic outcomes also carry significant value in this young patient population.

Future research directions should include prospective randomized trials comparing this combined approach to emerging therapies such as biological agents and targeted immunomodulators (28). Investigation of optimal treatment schedules, including maintenance protocols for preventing late recurrences, could further improve outcomes. Biomarker studies identifying patients most likely to benefit from specific treatment combinations would advance personalized medicine approaches (29).

The integration of traditional medicine concepts with modern technology exemplified by this study represents a broader trend in integrative medicine. Similar combined approaches have shown promise in other inflammatory conditions, suggesting potential applications beyond GLM (30). The success of this protocol may encourage further investigation of synergies between conventional and traditional therapies in complex inflammatory diseases.

Implications for clinical practice include reconsidering first-line therapy recommendations for GLM. Current guidelines emphasize stepwise escalation from observation to systemic therapy, often resulting in prolonged morbidity and multiple treatment failures (31). Our results suggest that early intervention with the combined approach could prevent disease progression, reduce overall treatment duration, and improve long-term outcomes while minimizing adverse effects.

## 5 Conclusion

The combination of acupoint glucocorticoid injection with focused ultrasound therapy appears to be a promising approach in GLM management, achieving high complete response rates of 92.2% with low recurrence (7.8%) and favorable safety profiles. This innovative integration of traditional Chinese medicine principles with modern interventional technology addresses key limitations of conventional therapies, offering superior efficacy, reduced systemic toxicity, and improved cosmetic outcomes. The approach particularly benefits young women of reproductive age who comprise the primary GLM demographic, providing effective treatment while preserving fertility potential and quality of life. These findings support considering this combined protocol as a first-line treatment option for GLM, though prospective validation through randomized controlled trials is warranted. The success of this integrative approach encourages further exploration of synergies between traditional and modern medicine in managing complex inflammatory breast diseases.

## Data Availability

The original contributions presented in the study are included in the article/Supplementary Material, further inquiries can be directed to the corresponding author.
